# Integrated Antitumor Activities of Cellular Immunotherapy with CIK Lymphocytes and Interferons against KIT/PDGFRA Wild Type GIST

**DOI:** 10.3390/ijms231810368

**Published:** 2022-09-08

**Authors:** Erika Fiorino, Alessandra Merlini, Lorenzo D’Ambrosio, Ilaria Cerviere, Enrico Berrino, Caterina Marchiò, Lidia Giraudo, Marco Basiricò, Annamaria Massa, Chiara Donini, Valeria Leuci, Ramona Rotolo, Federica Galvagno, Letizia Vitali, Alessia Proment, Soldano Ferrone, Alberto Pisacane, Ymera Pignochino, Massimo Aglietta, Giovanni Grignani, Giulia Mesiano, Dario Sangiolo

**Affiliations:** 1 Candiolo Cancer Institute, FPO-IRCCS, Strada Provinciale 142 Km 3.95, 10060 Candiolo, TO, Italy; 2 Department of Oncology, University of Turin, Regione Gonzole 10, 10043 Orbassano, TO, Italy; 3 Medical Oncology, Azienda Ospedaliera Universitaria S. Luigi, Regione Gonzole 10, 10043 Orbassano, TO, Italy; 4 Department of Medical Sciences, University of Turin, Corso Dogliotti, 14, 10126 Torino, TO, Italy; 5 Division of Surgical Oncology, Department of Surgery, Massachusetts General Hospital, Harvard Medical School, Boston, MA 02114, USA; 6 Department of Clinical and Biological Sciences, University of Turin, Regione Gonzole 10, 10043 Orbassano, TO, Italy

**Keywords:** immunotherapy, cytokine-induced killer cells, GIST

## Abstract

Gastrointestinal stromal tumors (GISTs) are rare, mesenchymal tumors of the gastrointestinal tract, characterized by either KIT or PDGFRA mutation in about 85% of cases. KIT/PDGFRA wild type gastrointestinal stromal tumors (wtGIST) account for the remaining 15% of GIST and represent an unmet medical need: their prevalence and potential medical vulnerabilities are not completely defined, and effective therapeutic strategies are still lacking. In this study we set a patient-derived preclinical model of wtGIST to investigate their phenotypic features, along with their susceptibility to cellular immunotherapy with cytokine-induced killer lymphocytes (CIK) and interferons (IFN). We generated 11 wtGIST primary cell lines (wtGISTc). The main CIK ligands (MIC A/B; ULBPs), along with PD-L1/2, were expressed by wtGISTc and the expression of HLA-I molecules was preserved. Patient-derived CIK were capable of intense killing in vitro against wtGISTc resistant to both imatinib and sunitinib. We found that CIK produce a high level of granzyme B, IFNα and IFNγ. CIK-conditioned supernatant was responsible for part of the observed tumoricidal effect, along with positive bystander modulatory activities enhancing the expression of PD-L1/2 and HLA-I molecules. IFNα, but not In, had direct antitumor effects on 50% (4/8) of TKI-resistant wtGISTc, positively correlated with the tumor expression of IFN receptors. wtGIST cells that survived IFNα were still sensitive to CIK immunotherapy. Our data support the exploration of CIK immunotherapy in clinical studies for TKI-resistant wtGIST, proposing reevaluation for IFNα within this challenging setting.

## 1. Introduction

Gastrointestinal stromal tumors (GISTs) are the most common malignant mesenchymal tumor of the gastrointestinal tract [1,2,3,4,5,6]. About 85% of GISTs are characterized by either *KIT* or platelet-derived growth factor receptor α (*PDGFRA*) mutations [4,5,6,7,8,9,10]. These can be effectively targeted with tyrosine kinase inhibitors (TKI) approved as different lines of treatment, i.e., imatinib, sunitinib, regorafenib and, in some countries, ripretinib (as first-, second-, third- and fourth-line treatment for advanced GISTs, respectively) [1].

Approximately 15% of GISTs carry neither *KIT* nor *PDGFRA* mutations. These tumors, historically called *KIT*/*PDGFRA* “wild type” GISTs (wtGISTs), represent an unmet diagnostic and therapeutic need and constitute an “umbrella” under which exceedingly rare disease sub-groups with peculiar molecular alterations and genetically so-far uncategorized GISTs are grouped together. In fact, the KIT/PDGFRA WT GIST classification gathers entities as different as proto-oncogene B-Raf (BRAF)-, neurofibromatosis type-1(NF1)-mutated, fibroblast growth factor receptor 1 (FGFR1)-, Neurotrophic Receptor Tyrosine Kinase (NTRK)-rearranged and succinate dehydrogenase (SDH)-deficient GISTs, as well as a number of KIT/PDGFRA WT GISTs for which no targetable oncogenic driver has been detected yet [1,11,12].

Currently, crucial and open research issues are the exploration of biological/immunological features and potential novel therapeutic vulnerabilities of wtGISTs. The development of hypothesis-driven strategies for wtGISTs requires reliable translational research models, to provide rational knowledge and the basis for clinical studies.

Indeed, the development of immunotherapy has provided a host of opportunities to match novel targets with innovative therapeutic strategies in many cancer types, and GISTs have been no exception to the rule [13,14,15,16]. Clinical studies have been developed to investigate immune checkpoint inhibitors (ICI) activity in GISTs combined with TKI, but none of them managed to show evidence of synergism [17,18]. This is possibly due to the fact that imatinib hampers type I IFN production and can decrease HLA-I expression on tumor cells [19], with a potentially detrimental role on the efficiency of ICI due to HLA-I expression reduction [20]; what is more, imatinib can alter intratumoral CD8^+^ T-cell subtype composition and activity [21]. Recently, the results of ICI monotherapy with nivolumab vs. nivolumab + ipilimumab in advanced GISTs patients, at least resistant/refractory to imatinib, have been published [22]: the primary endpoint (response rate > 15%) was not met, neither for nivolumab nor for nivolumab + ipilimumab. Hence, disappointingly, in spite of the strong biological rationale, immunotherapy has failed to provide significant survival benefits for advanced GISTs patients so far.

Within this scenario, we set a patient-derived preclinical model with wtGIST to explore a novel approach based on cellular immunotherapy with cytokine-induced killer lymphocytes (CIK), integrated with a re-evaluation of the composite antitumoral role of interferons (α and γ) within this setting. CIK are ex vivo expanded T-NK lymphocytes endowed with intense HLA-independent antitumor activity, which has already proved effective in several preclinical reports against various types of solid tumors including sarcomas and GISTs [23,24,25,26].

Exploiting HLA-independent cellular therapy allows us to address some main limitations that characterize tumor settings not responsive to checkpoint inhibitors, such as cold microenvironments with poor lymphocyte infiltration and/or reduced or defective antigen presentation. This peculiarity is particularly attractive in wtGISTs that have been reported to present less prominent immune infiltrate compared to KIT-mutated GISTs [27], which in turn present fewer immune infiltrating cells with respect to PDGFRA-mutated GISTs [28].

Besides the direct tumor killing activity, cellular immunotherapy with CIK may provide indirect beneficial effects, inflaming the tumor microenvironment and supplying important cytokines, such as type I interferons (IFNs). The potential antitumor activities of IFNs are of particular interest and should be re-evaluated, especially in the context of combinatorial strategies that might exploit their immunomodulatory effects. Evidence of such activities, in combination with a favorable safety profile, has been reported for IFNα in a phase II study for patients with advanced GISTs (NCT00585221) [29,30,31,32]. Here we focused on both CIK activity and the direct cytotoxic and indirect modulatory effects of IFNs within our wtGIST model, to highlight mechanistic insights and therapeutic opportunities to be envisioned in the context of combinatorial strategies for GISTs not responsive to targeted therapies.

## 2. Results

### 2.1. Generation and Characterization of Primary Cell Lines from Patients with Diagnosis of KIT/PDGFRA WT GIST

**Figure 1:**
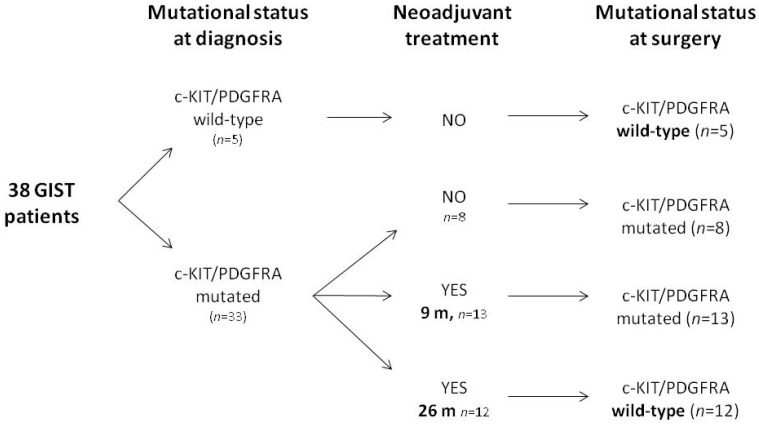
Schematic summary of the experimental platform from GIST patients. m = months.

We set a preclinical experimental platform based on primary cell lines derived from patients with a pathologic diagnosis of *KIT/PDGFRA* wtGIST. A study schematic of the experimental workflow is provided in Supplementary Figure S1. Mutational analysis of 38 GIST patients at diagnosis (*n* = 19 affected by advanced GIST and *n* = 19 with localized disease) identified *KIT/PDGFRA* mutations in 33 patients (87%), while the remaining 5 resulted *KIT/PDGFRA* WT (13%). Twenty-five out of the 33 patients with *KIT/PDGFRA*-mutated GIST at diagnosis underwent at least one line of systemic treatment with imatinib, sunitinib—or both —before surgery. Among these, in 13 out of the 25 patients the same mutational status detected at diagnosis was found after analysis of the surgical specimen while, in the remaining 12 patients’ cases, the surgical specimen (although showing adequate cellularity and by selecting only vital areas) resulted *KIT/PDGFRA* WT. The latter 12 patients had received significantly longer treatment courses (mean treatment duration: 26 months) with respect to cases in which the mutation was maintained (mean treatment duration: 9 months) (Figure 1).

**Figure 2:**
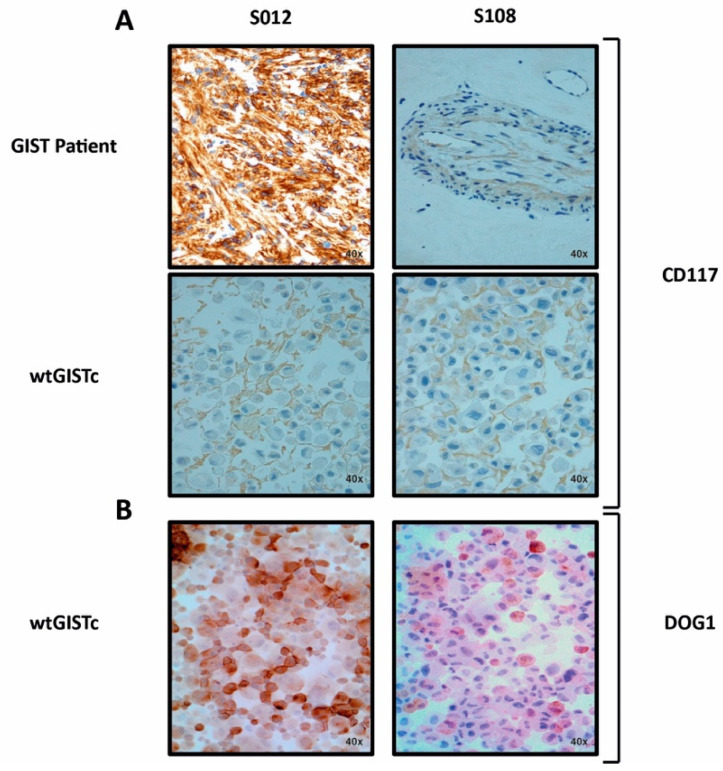
CD117 and DOG1 expression in GIST samples and corresponding wtGISTc. CD117 (**A**) and DOG1 (**B**) immunohistochemistry staining (magnification 40×) in two representative surgical samples from GIST patients (diffuse expression on the left, focal expression on the right) and the corresponding wtGISTc. S012 and S108: unique patient numbers.

We confirmed by pathology evaluation that the *KIT/PDGFRA* WT GIST samples had positive immunostaining for KIT (CD117)/DOG1 (Figure 2) and did not display dedifferentiation features. From this cohort, we successfully generated 11 primary cell lines of *KIT/PDGFRA* WT GIST (wtGISTc). Primary cell lines showed morphological features and IHC patterns consistent with the pathology evaluation of the corresponding tumors (Figure 2).

We confirmed the genetic consistency between wtGISTc (*n* = 11) and their original surgical samples by DNA sequencing. All wtGISTc resulted *KIT/PDGFRA* WT; additional genetic alterations, detected by using a targeted panel of 523 cancer-related genes, are shown in Table 1.

As further characterization, we confirmed the conserved HLA-I membrane expression by our wtGISTc. We reported the expression rate for the inhibitory immune-checkpoints molecules PD-L1 (38 ± 7%) and PD-L2 (28 ± 6%), as shown in Supplementary Figure S2, along with the main target molecules (NKG2D ligands) recognized by CIK lymphocytes (ULPBPs 2/5/6 = 59 ± 4% and MICA A/B = 15 ± 5%)). A negligible expression was detected for the DNAM-1 ligands, CD112 and CD155. The tumorigenic potential in vivo of wtGISTc was confirmed (*n* = 8) in immunodeficient mice, with a mean time of engraftment of 6 weeks.

### 2.2. wtGISTc Are Resistant to Imatinib and Moderately Sensitive to Sunitinib

**Figure 3:**
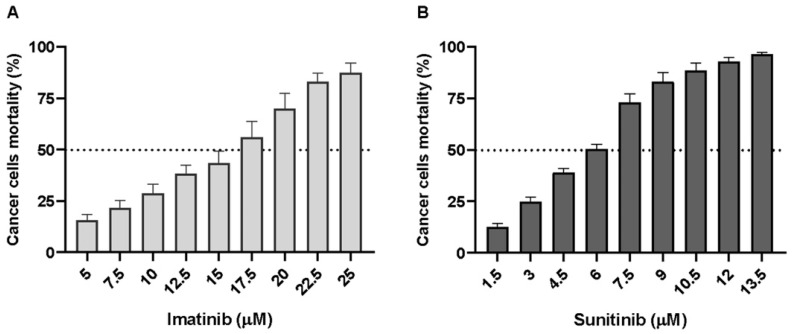
Sensitivity to imatinib and sunitinib of wtGISTc in vitro. wtGISTc resulted resistant to imatinib (IC50 17 ± 8 µM, mean ± SEM) (**A**) and moderately sensitive to sunitinib (IC50 6 ± 2 µM, mean ± SEM) (**B**). A498 RCC line was used as a positive control of sunitinib sensitivity.

We explored in vitro the sensitivity of wtGISTc to imatinib and sunitinib. All the wtGISTc (*n* = 11) were resistant to imatinib. Treatment of wtGISTc with high doses of imatinib, ranging from 5 to 25 µM, resulted in a mean IC50 dose of 17 ± 8 µM (*n* = 20 Figure 3A). wtGISTc resulted only moderately sensitive to sunitinib (*n* = 37, Figure 3B) with a mean IC50 dose of 6 ± 2 µM, if compared to sensitive control (A498 RCC line, IC50 5 µM).

### 2.3. Generation and Characterization of CIK Lymphocytes from GIST Patients

**Figure 4:**
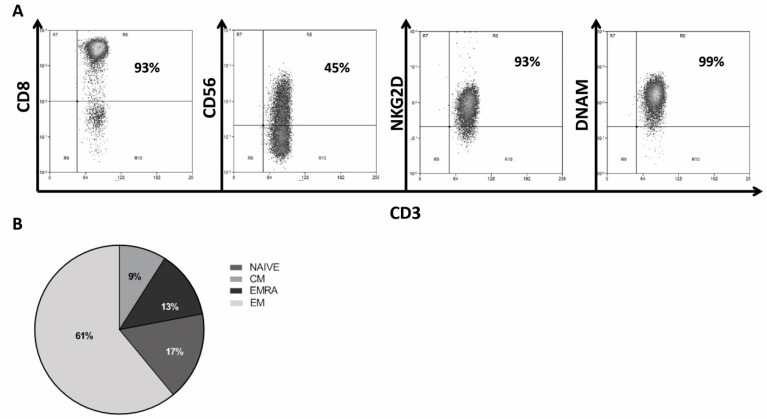
Phenotype and main subsets of patient-derived CIK lymphocytes. Representative phenotype of CIK at the end of their ex vivo expansion; mature CIK were mainly CD3^+^CD8^+^ with a relevant CD3^+^CD56^+^ double-positive subset. The receptors mostly imputed to cancer cell recognition, NKG2D and DNAM-1, were expressed on a high percentage of cells (**A**). Distribution of main lymphocyte subsets with effector memory (EM), effector memory-RA^+^ (EM RA), central memory (CM), and naïve (**B**).

CIK were successfully generated and expanded ex vivo starting from PBMC of 13 patients with GIST. The median expansion fold was 184 (31–1258), a good percentage of mature CIK was CD3^+^CD56^+^ with a median value of 43% (24–60). Mature CIK were mainly CD8^+^ with a median value of 81% (64–91). As expected, the receptors NKG2D and DNAM-1 were highly expressed by mature CIK with a median value of 86% (40–92) and 94% (86–100), respectively. The percentage of NK cells in the bulk CIK population was negligible. Representative dot plots with phenotype of mature CIK are reported in Figure 4A. At the end of the ex vivo expansion, the different lymphocyte subsets in bulk CIK (*n* = 13) were the effector memory (EM: CD62L^−^CD45RA^−^ 61 ± 3%) (Figure 4B, *n* = 32). We observed a low membrane expression of PD-1^high^ (4 ± 2%), and LAG3^high^ (5 ± 1%) expression on a low percentage of cells, while TIM3 (96 ± 1%), and TIGIT (92 ± 1%) were expressed on a high percentage of cells, as shown in Supplementary Figure S3.

### 2.4. CIK Are Capable of Direct and Indirect Cancer Cell Killing and Immunomodulation in wtGISTc Resistant to TKI

**Figure 5:**
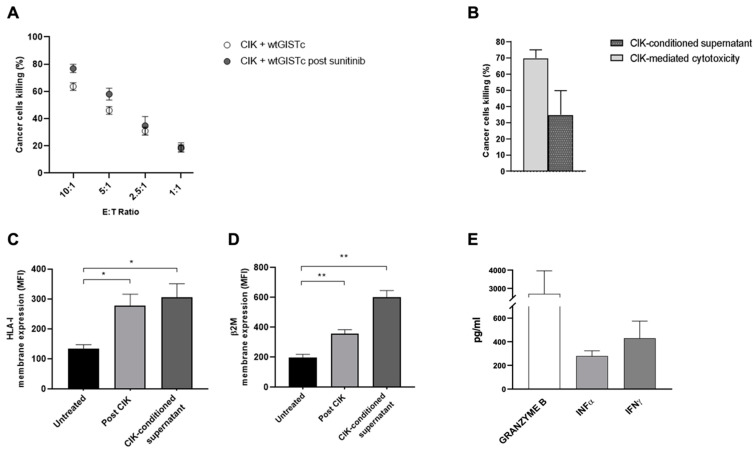
(**A**) Antitumor activity of CIK against wtGISTc resistant to TKI. Patient-derived CIK efficiently killed in vitro wtGISTc resistant to imatinib. The CIK killing ability was retained when sequentially tested against wtGISTc that survived treatment with sunitinib. (**B**) CIK-conditioned medium is endowed with tumoricidal activity against wtGISTc in vitro. CIK-conditioned supernatants, collected after 72 h co-culture of CIK with wtGISTc, was shown to be capable of cancer cell killing activity even in the absence of a direct CIK-tumor contact. (**C**,**D**) Direct and indirect modulation of HLA-I and β2M in wtGISTc by CIK lymphocytes. The membrane expression of both HLA-I (**C**) and β2M (**D**) on wtGIST was enhanced after treatment with CIK or CIK-conditioned medium (* *p* < 0.05; ** *p* < 0.005, nonparametric *t*-test). (**E**) Soluble factors produced by activated CIK lymphocytes. Granzyme B, IFNα and IFNγ are intensely produced by CIK following contact with wtGIST (10:1 E:T).

We explored the cytotoxic activity of patient-derived CIK (*n* = 12) against wtGISTc (*n* = 9) resistant to imatinib. In 4/9 cases we were able to set up an autologous match as the target (wtGISTc) and the effectors (CIK) were derived from the same patients. CIK efficiently killed wtGISTc, with a specific cancer cell lysis of 62 ± 2.3%, 45 ± 3.1%, 30 ± 3.2% and 18 ± 2.8% at progressively decreasing effector/target ratios (10:1, 5:1, 2.5:1 and 1:1, respectively). In selected experiments (*n* = 4) we confirmed that CIK retained their full antitumor potential even when sequentially tested against wtGIST cells that survived treatment with sunitinib in vitro (IC50 dose, Figure 5A). Besides the direct CIK-mediated cytotoxicity, we questioned and explored the indirect activities that CIK may exert against wtGISTc through the secretion of soluble factors, intended either as tumoricidal or immunomodulatory effects. First, we observed that CIK-conditioned supernatant, collected at the end of cancer cell killing assays, was capable of killing wtGISTc (mean cancer cell specific mortality 35 ± 2%) (Figure 5B). Furthermore, CIK-conditioned medium also significantly enhanced HLA-I (2-fold) and β2-microglobulin (B2M)-2.7-fold) membrane expression on wtGISTc (*n* = 4) (Figure 5C,D). Consistently, we observed the enhanced HLA-I/B2M expression also in the residual wtGIST cells collected at the end of the cytotoxicity assays with CIK lymphocytes, supporting a potentially relevant bystander immunomodulatory effect following CIK cellular immunotherapy. The analysis by ELISA of the CIK-conditioned medium, always collected at the end of 72 h cancer cell killing assays (*n* = 6), confirmed an intense production of protease granzyme B along with IFNα and IFNγ (Figure 5E).

### 2.5. Antitumor and Immunomodulatory Activity of IFNα and IFNγ in wtGISTc

**Figure 6:**
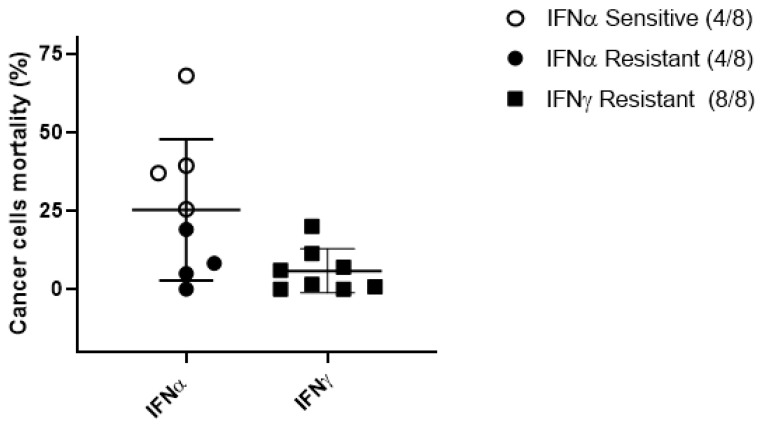
Sensitivity of wtGISTc to IFNα and IFNγ. After treatment, all wtGISTc resulted resistant to IFNγ and 4 out of 8 wtGISTc were relatively sensitive to IFNα (mean tumor cell death 42%).

While granzyme B was expected as one of the main mediators of CIK cytotoxicity, we focused on the observed presence of IFNs, as these cytokines may be of particular relevance in this context, considering their past empirical consideration in GIST clinical studies [29]. We assessed the tumoricidal and immunomodulatory effects of IFNα and IFNγ on our wtGISTc resistant to TKI. IFNα (1 × 10^4^ IU/mL) was capable of direct cancer cell cytotoxicity in 4/8 (50%) of wtGISTc tested, while no significant cancer cell cytotoxicity was mediated by IFNγ (1 × 10^3^ IU/mL) (Figure 6).

**Figure 7:**
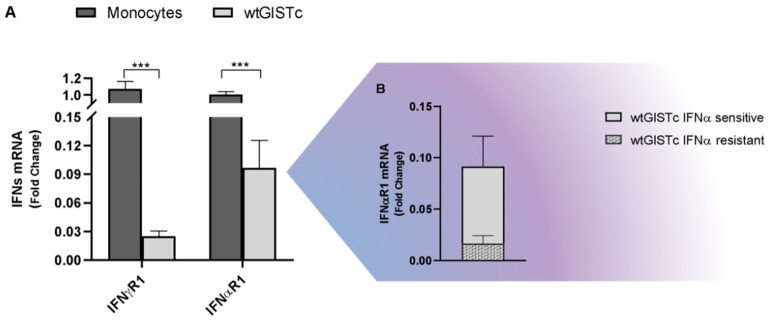
IFNα and IFNγ receptors (subunit 1) in wtGISTc. wtGISTc were confirmed to express IFNα/ɣ R1 even if at a lower level than monocytes, used as positive controls (**A**). The expression levels of IFNαR1 mRNA were significantly higher in wtGISTc sensitive to IFNα (**B**). All values are represented in fold change and normalized on three housekeeping genes (GAPDH, HPRT and PGK). (*** *p* < 0.001, nonparametric *t*-test).

Both IFN-sensitive and resistant wtGISTc retained their in vitro susceptibility to CIK mediated killing, as demonstrated by sequential treatments (not shown). We confirmed by qRT-PCR the expression of IFNαR1 and IFNγR1 in wtGISTc (*n* = 8), observing their higher levels among wtGISTc sensitive to IFNα (Figure 7B).

**Figure 8:**
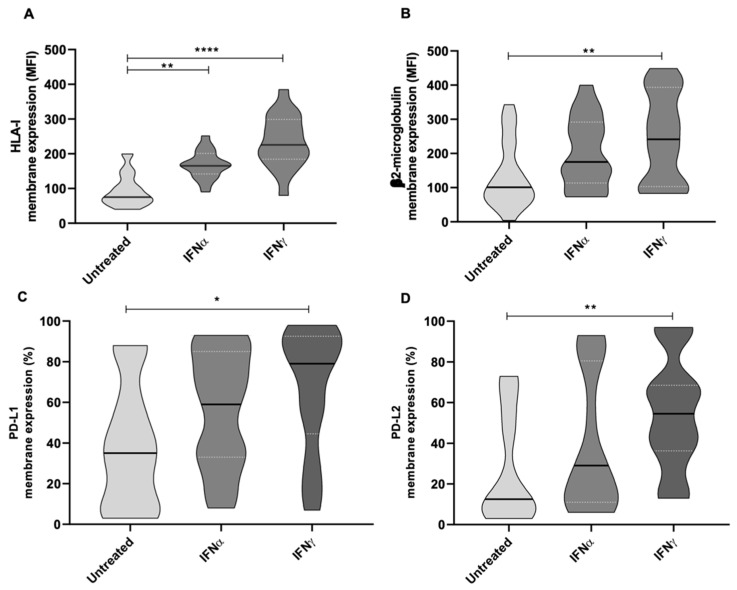
Modulation of HLA-I, β2-microglobulin, PD-L1 and PD-L2 expression mediated by IFNs in wtGISTc. IFNs determined a significant enhancement in the expression of HLA-I/B2M and immune checkpoints (PDL-1/2) in wtGISTc. All the reported values were compared to untreated controls and expressed in MFI. (**** *p* < 0.0001; ** *p* < 0.005; * *p* < 0.05; nonparametric *t*-test).

Along with the reported direct tumoricidal effect, we observed that treating wtGISTc (*n* = 8) in vitro with both IFNα and IFNγ resulted in potentially relevant immunomodulatory effects. Namely, we observed a significantly enhanced membrane expression of HLA-I/B2M molecules and immune checkpoints PD-L1 and PD-L2 (Figure 8A–D).

## 3. Discussion

In our study we explored and report the preclinical vulnerability of *KIT/PDGFRA* WT GISTs to cellular immunotherapy with CIK lymphocytes, highlighting potentially relevant antitumor and immunomodulatory effects that prompt new considerations on the role of IFNs in this challenging setting.

Studying the peculiar biological features of wtGIST, along with the exploration of effective therapeutic interventions, requires the availability of reliable investigational/experimental models. To this end, our patient-derived platform provides a reliable approximation of realistic scenarios, allowing the acquisition of new, important immune-profiling information on wtGIST and explorative therapeutic opportunities. As a first consideration, the observed prevalence of wtGIST, either primitive or emerging after prolonged imatinib treatment, supports clinical reflections on the opportunity to repeat biopsies to reevaluate the molecular profiling of GIST at the time of disease progression. Small case series have reported KIT expression loss after imatinib treatment [33]; nonetheless, whether mutational re-testing is necessary at every surgical intervention/at every disease progression is not yet defined, even in international guidelines. Second, the preserved expression of HLA-I molecules on wtGIST cells, and their upregulation upon treatment with CIK or IFNs, is indicative of an indirect positive immunomodulatory effect of such treatments that could trigger activities from the adaptive immune response [20].

It is important to note that the in vitro dose of IFNα used in our experiments (1 × 10^4^ IU/mL) is a much higher concentration as compared with the reported spontaneous CIK production that is, however, still capable of inducing immunomodulatory effects. Considering the future design of clinical trials in combination with cellular immunotherapy, it is conceivable that different and even lower ranges of IFNα may be tested with the intent of mainly exploiting its immunomodulatory effect while reducing potential toxicities.

The modulatory activity exerted by IFNs is associated with the expression of regulatory immune checkpoints (PD-L1/2), as confirmed by our data. On one side, this could contribute to the immune escape of wtGIST, as recently reported by Vitiello et al. [28], but also set a conceptual frameshift to envision possible synergisms with checkpoint inhibitors [34,35,36]. Finally, the phenotypic profiling of wtGIST confirms the proficient expression of stress-inducible molecules that mechanistically substantiate the observed activity of CIK lymphocytes and underscores the potentialities, either endogenous or therapeutically induced, of effectors from the innate immune system in this challenging setting.

In previous works, we reported the intense activity of CIK against multiple types of soft tissue sarcomas, including GIST [23]. The relevance of the present report is the evidence that patient-derived CIK retain their potentialities even within the peculiar wtGIST setting, resistant to TKIs. The observed tumoricidal effects by CIK against wtGIST cells that survived a pretreatment with either sunitinib or IFNs underscore their potential role in the treatment of drug-resistant *KIT/PDGFRA* WT GISTs.

A possible limitation may be seen in the fact that our study did not include in vivo experiments. In previous works, we reported the pharmacodynamic properties and tumor recruitment capabilities of CIK lymphocytes in vivo. A tumor xenograft model in immunodeficient mice would not add much substance to the main core findings of the present study, where the exploration of the integrated direct and indirect immunomodulatory activities of CIK and IFNs would require a complex immune-competent model. Based on the described preclinical data, the best next experimental steps should be probably designed within controlled clinical studies that could benefit from the already existing knowledge of previous trials with IFNα in GIST patients, and from the safety and pharmacodynamic information with CIK in various tumor settings. Our findings highlight and propose also a new perspective on the therapeutic role of IFNα in wtGISTs that are resistant (or progressed) to conventional TKI treatments. We provide experimental evidence that IFNα may exert a direct cytotoxicity on wtGIST cells, resistant to imatinib, along with important immunomodulatory effects that could reactivate the individual adaptive immune response or offer opportunities for synergism with checkpoint inhibitors, as ongoing trials are currently exploring.

Of note is that the observed positive correlation between the membrane expression of IFNR1 on wtGIST and their sensitivity to IFNα may raise both biological and clinical considerations. On one side, it could have a predictive role while, on the other one, it provides a rationale to explore molecular strategies that could restore its expression.

Overall, our findings highlight the need for the molecular re-evaluation of GIST at disease progression and support the exploration of CIK cellular immunotherapy in clinical studies for patients with TKI-resistant, *KIT/PDGFRA* wild type GIST. A reappraisal and reinterpretation of the role of IFNα, either as monotherapy or in the context of integrated immunotherapy approaches, emerges and warrants clinical consideration.

## 4. Materials and Methods

### 4.1. Experimental Platform and Generation of Wild-Type Primary GIST Cell Lines

Our tumor samples, primary cell lines and cultures were generated from biopsies obtained from patients with confirmed diagnoses of GIST. All patients gave informed consent for the collection and use of their biological material for research purposes, approved by the ethics committee. All surgical samples were analyzed for c-KIT (exons 9,10,11,13 and 17) and PDGFRA (exons 12,14 and 18) mutations. Primary wild-type GIST cultures (wtGISTc) were generated, starting from surgical samples, by mechanical (scalpel) dissection and enzymatic digestion with type I collagenase (Invitrogen, Carlsbad, CA, USA) or by the gentleMACS instrument (Miltenyi Biotec, Bologna, Italy) using the specific human tumor dissociation kit. Tumor cells were resuspended in Iscove’s Modified Dulbecco’s Medium (IMDM, Sigma Aldrich, Saint Louis, MO, USA) with 15% heat-inactivated Fetal Bovine Serum (Euroclone Spa, Milan, Italy), penicillin and streptomycin (100 U/mL, Sigma Aldrich, Saint Louis, MO, USA), L-glutamine (2 mM, Sigma Aldrich, Saint Louis, MO, USA) and seeded in plates treated for anchorage-dependent cultures (Corning/Costar, VWR International PBI s.r.l., Milan, Italy) at standard conditions of 37 °C and 5% CO_2_. All cell cultures obtained were analyzed for c-KIT (exons 9,10,11,13 and 17) and PDGFRA (exons 12, 14 and 18) mutations by Real-time qPCR (RT-qPCR) to confirm the conformity with the mutational status of original surgical samples. In vivo assays were conducted to confirm the tumorigenicity of wtGISTc: 1 × 10^6^ tumor cells re-suspended in 200 μL of a 1:1 mix of PBS 1× and Matrigel Basement Membrane Matrix (Sigma Aldrich, Saint Louis, MO, USA) were implanted subcutaneously in six-week-old NOD/LtSz-scid/scid (NOD/SCID; Charles River Laboratories, Calco, Italy) female mice. Tumor growth was monitored weekly with calipers and mice were sacrificed when tumors reached a maximum main diameter of 2 cm.

### 4.2. CIK Cells Culture and Ex Vivo Expansion

CIK cells were expanded from fresh or cryopreserved peripheral blood mononuclear cells (PBMC) from 13 patients with confirmed GIST diagnoses. All patients gave informed consent for the collection and use of their biological material for research purposes, approved by the ethics committee. PBMC were separated by density gradient centrifugation (Lymphoprep, Aurogene s.r.l., Rome, Italy) and seeded into cell culture flasks (2 × 10^6^ cells/mL) in RPMI-1640 medium (Gibco BRL Life Technologies, Monza, Italy) supplemented with 10% heat-inactivated fetal bovine serum (Sigma Aldrich, Saint Louis, MO, USA), penicillin and streptomycin (100 U/mL, Gibco BRL Life Technologies, Italy), L-glutamine (2 mM, Sigma Aldrich, Saint Louis, MO, USA) at 37 °C and 5% CO_2_. IFNγ (1000 U/mL, Miltenyi Biotec, Bologna, Italy) was added on day 0; after 24 h, recombinant human interleukin IL-2 (300 U/mL, Miltenyi Biotec, Bologna, Italy) and anti-CD3 antibody (50 ng/mL, MACS Miltenyi Biotec, Bologna, Italy) were added. Cells were ex vivo expanded for 3 weeks. Fresh medium and IL-2 (300 U/mL) were added every 3 days, and the cell concentration was maintained at 2−1.5 × 10^6^ cells/mL.

### 4.3. Flow Cytometry

To evaluate phenotype features, wtGISTc and CIK were labeled and acquired on a FACS Cyan (CyAN ADP, Beckman Coulter s.r.l., Cassina de’ Pecchi, Italy). Flow cytometry data were analyzed using Summit Software (Beckman Coulter s.r.l., Cassina de’ Pecchi, Italy). wtGISTc were stained with the following fluorescein isothiocyanate (FITC), phycoerythrin (PE)-, or allophycocyanin (APC)-conjugated mouse monoclonal antibodies (mAbs): MICA/B and ULBP1, ULBP2, 5, 6 and ULPB3 for the NKG2D ligands (Pharmingen, Milan, Italy), CD112 and CD155 (R&D Systems, Minneapolis, MN, USA) for DNAM ligands and PD-L1 and PD-L2 (Pharmingen, Milan, Italy). HLA-I and β2-microglobulin (in collaboration with Soldano Ferrone, clones TP25.99.8.4 [37] and L368 [38], respectively), and Human IFNα/β R1 and IFNγ R1/CD119 Antibody (R&D Systems, Minneapolis, MN) were detected on wtGISTc with the use of a secondary antibody (Goat Anti-Mouse Ig, Pharmigen, Milan, Italy). Conjugated anti-human monoclonal antibodies for CD3 (Pharmingen, Milan, Italy), CD8, CD56, NKG2D, CD62L, CD45RA, PD-1, TIM-3 (MACS Miltenyi Biotec, Bologna Italy), DNAM-1, LAG3 (BD Biosciences, Milan Italy), TIGIT (eBiosciences, San Diego, CA, USA), NKp30, NKp44 and NKp46 (MACS Miltenyi Biotec, Bologna, Italy) were used to characterize CIK.

### 4.4. mRNA Analysis

Total RNA automatic extraction of wtGISTc was performed using the Maxwell RSC miRNA Tissue KIT and Maxwell RSC Instrument (Promega, Milan, Italy), according to the manufacturer’s instructions. The RNA was reverse-transcribed into cDNA by a High-Capacity cDNA Reverse Transcription Kit (Applied Biosystems by ThermoFisher Scientific, Rodano, Italy) and quantified with a Nanodrop spectrophotometer (The DeNovix DS-11 FX Series). cDNA was amplified by RT-qPCR using a SYBR^®^ Green Master Mix probe (Advanced Universal SYBR Green Master Mix, Biorad, Segrate, Italy). Specific primers for subunit 1 of IFNα and IFNγ receptor genes were used: IFNα receptor forward 5′-CATCACGTCATACCAGCCATTT-3′, reverse 5′-CTGGATTGTCTTCGGTATGCAT-3′; IFNγ receptor forward 5′-GCGCCTGTTGTCTTAGCTAC-3′, reverse 5′-CTCATCCAATGCAAGTCCGG-3′. Data analyses were conducted, comparing the expression of IFNα and IFNγ receptors in wtGISTc and monocytes (positive controls). The gene expression levels were normalized to three different housekeeping genes’ (GAPDH, PGK and HPRT) expression and reported as fold change. The monocytes were separated from the PBMC with human CD14 microbeads (MACS Miltenyi Biotec, Bologna, Italy), according to manufacturer’s instructions.

### 4.5. DNA Sequencing Analysis and Gene Mutation Profile

Genomic DNA for each sample underwent deep sequencing using the Illumina TruSight Oncology 500 panel (Illumina, Milan, Italy). The targeted panel was 1.94 Mb in size, encompassing the exon sequences of 523 cancer-related genes (coding size 1.2 Mb). The MSI status of approximately 120 loci and the tumor mutation burden were also assessed by the panel. Following the manufacturer’s protocol, the DNAs were sonicated using the Covaris Focused-ultrasonicator (Covaris, Woburn, MA, USA), the UMIs adapted and then indexed. Two rounds of hybrid-capture allowed an enrichment of the targeted sequence. The libraries were then sequenced on the Illumina-NovaSeq 6000 instrument (Illumina, Milan, Italy) to reach a minimum of 500× read depth. Raw data were processed by the Illumina Local App associated with the TSO500 panel (TruSight Oncology 500 v2.2 Local App) to produce FASTQ files through the alignment of the sequence to the human reference sequence GRCh37 (hg19). The Local App also performed sequencing QCs and somatic variant-calling with a tumor-only pipeline. We confirmed the mutational status for both KIT and PDGFRA genes using the mass spectrometry matrix-assisted laser desorption ionization time-of-flight method (Maldi-TOF) on the MassARRAY System (Agena Bioscience, San Diego, CA, USA) with the Myriapod^®^ GIST primer set (cod. SQ030) (Diatech Pharmacogenetics, Jesi, Italy). A small aliquot of DNA was amplified with the specific primer set encompassing the *KIT* and *PDGFRA* regions covered by the panel. After the purification of the PCR products, a single base extension was applied, and the mass of the single-based extended primers was checked with the Maldi-TOF method. Mass spectra were analyzed with the MassARRAY^®^ Analyzer 4 software (Agena Bioscience, Lincoln, NE, USA).

### 4.6. In Vitro Drug Sensitivity and Cancer Cell Killing Assays

To assess the sensitivity of wtGISTc to target therapy and interferons’ direct cytotoxic activity, tumor cells were seeded in 96-multiwell plates (Corning/Costar, VWR International PBI s.r.l., Milan, Italy). After 24 h, the cells were treated with scalar doses of imatinib (Selleckem, Houston, TX, USA) from 5 µM to 25 µM, sunitinib (Selleckem, Houston, TX, USA) from 0,1 µM to 15 µM, IFNα (1 × 10^4^ IU/mL, Myltenyi Biotec, Bologna, Italy) or IFNγ (1 × 10^3^ IU/mL, Peprotech, Neuilly-sur-Seine, France). The A498 renal cancer carcinoma (ACC) cell line was used as the control for sunitinib sensitivity (IC50 5 µM). To study the therapeutic combination between the imatinib/sunitinib and the IFNα/IFNγ, wtGISTc were seeded in 96-multiwell plates (Corning/Costar, VWR International PBI s.r.l., Milan, Italy) in triplicate and, after 24 h, were treated with imatinib (therapeutic dose 25 nM), sunitinib (IC50 dose) IFNα (1 × 10^4^ IU/mL) and IFNγ (1 × 10^3^ IU/mL) alone or in combination. The tumor cell lysis was assessed with the CellTiter-Glo^®^ Luminescent Cell Viability Assay (Promega, Milan, Italy), according to the manufacturer’s instructions. The luminescence signal was determined with a microplate reader (Multi-Mode microplate Readers Biotek SRL, Milan, Italy), indicating the number of viable and metabolically-active target cells by quantifying the ATP. The IC50 doses (imatinib and sunitinib) correspond to IC values ranging between 40% and 60%. The wtGISTc were considered IFNs-sensitive if the mortality after exposure resulted greater or equal to 25%; otherwise, they were considered resistant. The percentages of tumor lysis were evaluated after 72 h of each treatment. The wtGISTc treated with an equal volume of cell culture medium alone was used as the control. To assess IFNα and IFNγ immunomodulatory activity, wtGISTc were seeded in 6-multiwell plates and after 24 h, treated with IFNα (1 × 10^4^ IU/mL, Myltenyi Biotec, Bologna, Italy) or IFNγ (1 × 10^3^ IU/mL, Peprotech, Neuilly-sur-Seine, France) for 72 h at 37 °C and 5% CO_2_. Tumor cells were detached with Stem Pro Accutase Cell Dissociation Reagent (ThermoFisher Scientific, Waltham, MA, USA) and stained with mAb for flow cytometry, as previously described. We assessed the tumor-killing abilities of patient-derived CIK in vitro against wtGISTc. Cytotoxicity assays against the wtGISTc were performed using flow cytometry or a bioluminescent cell viability assay. For the flow cytometry assays, the target cells were stained with the vital dye PKH26 kit (Sigma-Aldrich, Saint Louis, MO, USA), according to the manufacturer’s protocol. The immune-mediated killing was analyzed with flow cytometry (Cyan ADP, Dako, Milan, Italy) and measured by the DAPI permeability of the target cells (PKH26^+^ gate). In selected experiments, immune-molecules modulation on the wtGISTc by CIK was evaluated by flow cytometry. For the bioluminescent method, the cytotoxicity was measured with the CellTiter-Glo^®^ Luminescent Cell Viability Assay (Promega, Milan, Italy), previously described. CIK cells were co-cultured at different effector:target ratios (10:1, 5:1, 2.5:1 and 1:1) in cytotoxicity assays (300 U/mL IL-2 medium at 37 °C and 5% CO_2_) for 72 h. In selected experiments, we tested CIK cytotoxicity against the wtGISTc after molecular targeted therapy and interferons exposure. To study the indirect cytotoxic activity, in selected experiments, the CIK-conditioned supernatant was collected at the end of the tumor killing assays at effector:target ratio 10:1. Indirect cytotoxicity assays of CIK-conditioned supernatant against the wtGISTc were performed using flow cytometry, after 72 h exposure. To assess the CIK-conditioned supernatant immunomodulatory activity, after 72 h, the wtGISTc were labeled with mAb and the immune molecules were evaluated by flow cytometry, as previously described. The internal control target cells were tested alone, separately from the CIK, to assess spontaneous mortality. The percentage of wtGISTc-specific lysis for each effector:target cell ratio was calculated using the following formula: [(experimental−spontaneous mortality/100−spontaneous mortality) × 100].

### 4.7. In Vitro Soluble Factors Production

To quantify the release of soluble factors, the CIK cells were co-cultured with tumor targets in RPMI with 300 U/mL IL-2 at a 10:1 effector:target ratio, incubated at 37 °C for 72 h and then the cell culture supernatant was collected. The Human Luminex Discovery Assay (R&D System, Milan, Italy) was used to measure the IFNα and Granzyme-B production. Data were acquired and analyzed by Bioclarma (Analysis Service). The IFNγ concentration was evaluated in the cell culture supernatant with the Human IFN-γ ELISA Kit (Diaclone, Besançon, France), according to the manufacturer’s instructions. Each sample was measured in duplicate and all data were analyzed and reported following their normalization on spontaneous cytokines production by effectors and targets alone.

### 4.8. Statistical Analysis

All the experiments were performed at a minimum as duplicates. The data were analyzed using the GraphPad Prism 8.0 (GraphPad Software, San Diego, CA, USA). Descriptive data are presented as mean ± SEM or median with related ranges. To find statistical significance in the comparison of two groups, we relied on two-tailed Student’s *t*-tests; for comparison of three or more groups, the data were analyzed by two-way ANOVA with Kruskal–Wallis test multiple comparisons. A *p*-value < 0.05 was considered significant. Significance is represented on graphs as * *p* < 0.05, ** *p* < 0.01, *** *p* < 0.001 and **** *p* < 0.0001.

## Figures and Tables

**Table 1:** Mutational profile of wtGISTc as assessed by targeted sequencing. VAF: Variant Allele Frequency.

wtGISTc	Gene Name	Variant Type	Single Nucleotide Variant ID	VAF (%)	Fold Change
**S012**	SPEN	inframe_deletion	chr1_16260673_CCTCACTGGTCTGGTGAGCGCA_C	27	
	LATS2	missense_variant	chr13_21562627_G_A	36	
	JAK2	amplification	chr9		1.946
	PTEN	amplification	chr10		1.445
**S061**	/	/	/	/	
**S075**	/	/	/	/	
**S108**	NOTCH1	missense variant:splice region variant	chr9_139401170_G_A	51	
**S125**	/	/	/	/	
**S148**	ARID1B	missense_variant	chr6_157100551_G_C	47	
	PRKDC	missense_variant	chr8_48805728_C_G	24	
**S173**	MYCN	missense_variant	chr2_16082319_C_T	20	
	GNAS	missense_variant	chr20_57474036_G_T	19	
**S188**	PGR	missense_variant	chr11_100998322_C_G	31	
**S220**	HIST3H3	missense_variant	chr1_228612992_G_A	16	
	LRP1B	missense_variant	chr2_141201961_G_T	19	
	CUL3	missense_variant	chr2_225379486_G_A	20	
	PRKDC	missense_variant	chr8_48866988_T_G	58	
	MALT1	missense_variant	chr18_56363602_A_G	16	
	NF2	stop_gained	chr22_30038226_C_A	50	
	FGFR1	amplification	chr8		1.533
	MYC	amplification	chr8		1.637
**S243**	IRS1	stop_gained	chr2_227661875_G_T	9	
	PDGFRB	inframe_deletion	chr5_149495390_AGCTCTG_A	7	
	NOTCH4	missense_variant	chr6_32189007_G_A	7	
**S288**	FGF2	missense_variant	chr4_123813429_C_T	51	
	FAT1	missense_variant	chr4_187628598_T_C	46	

## Data Availability

Data are available upon reasonable request.

## Supplementary Materials

The following supporting information can be downloaded at: https://www.mdpi.com/article/10.3390/ijms231810368/s1.
