# Supplementary material for: Integrated Antitumor Activities of Cellular Immunotherapy with CIK Lymphocytes and Interferons against KIT/PDGFRA Wild Type GIST
Source: Int J Mol Sci. 2022 Sep 8;23(18):10368. doi: 10.3390/ijms231810368 (PMC9499671; doi:10.3390/ijms231810368)
Supplement: Supplementary file 1 [file ijms-23-10368-s001.zip › ijms-1850516-supplementary.pdf]

Fiorino E. et al.

Integrated antitumor activities of cellular immunotherapy with CIK lymphocytes and interferons against KIT/PDGFRA wild type GIST.

Supplementary Materials

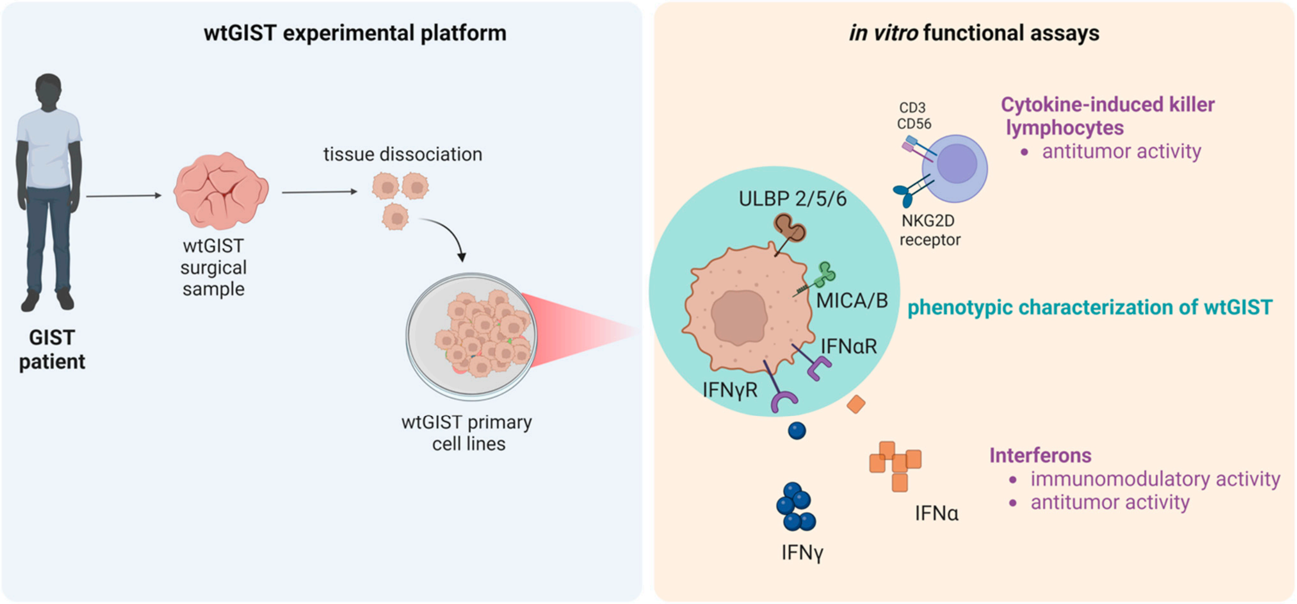

Figure S1. Study schematic with experimental platform for patient-derived cells (left) and *in vitro* functional assays (right). Created with BioRender.com.

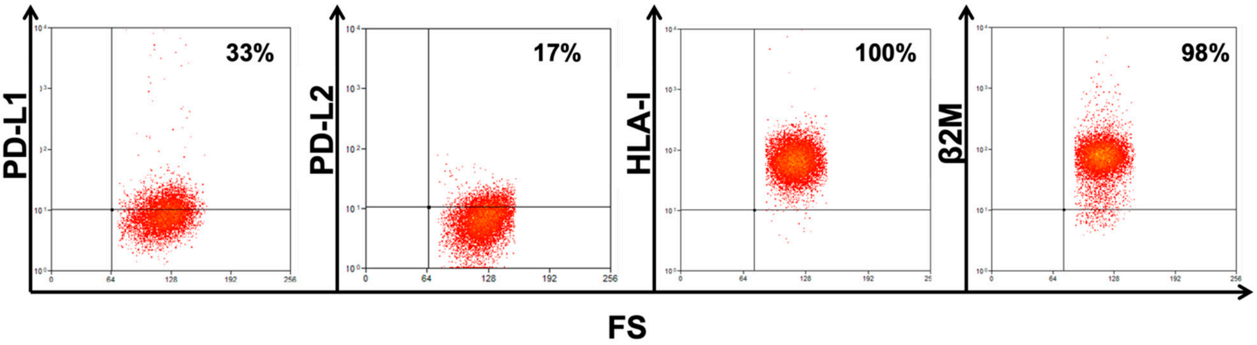

Figure S2: exemplificative cytometry dot plots showing PD-L1, PD-L2, HLA-I/ β2M on wtGISTc.

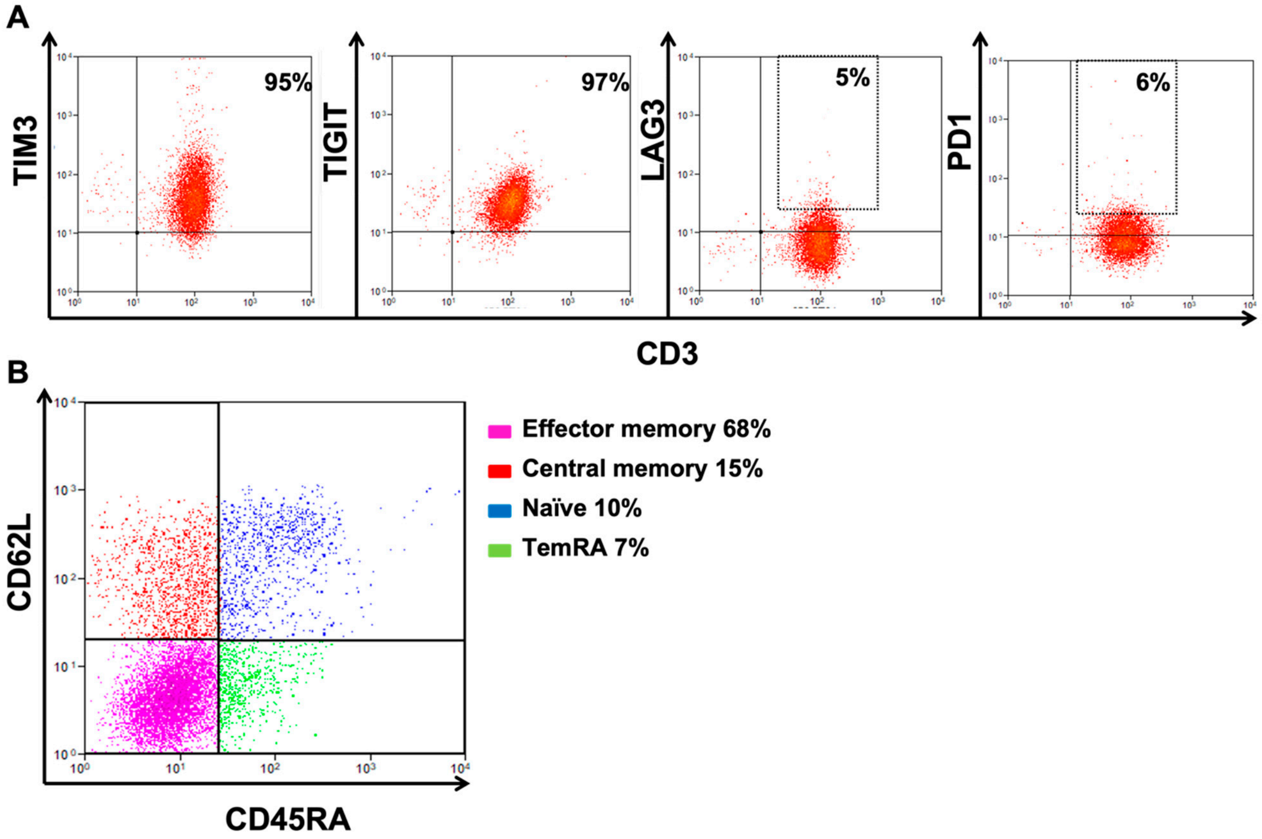

Figure S3: exemplificative cytometry dot plots showing CIK phenotype and immune checkpoints expression.
